# Micro- and Nano-vesicles from First Trimester Human Placentae Carry Flt-1 and Levels Are Increased in Severe Preeclampsia

**DOI:** 10.3389/fendo.2017.00174

**Published:** 2017-07-24

**Authors:** Mancy Tong, Qi Chen, Joanna L. James, Peter R. Stone, Lawrence W. Chamley

**Affiliations:** ^1^Department of Obstetrics and Gynaecology, School of Medicine, The University of Auckland, Auckland, New Zealand

**Keywords:** preeclampsia, trophoblast, particle, exosome, vesicle, vascular endothelial growth factor, endothelial dysfunction

## Abstract

**Background/objectives:**

Preeclampsia is a life-threatening hypertensive disease affecting 3–5% of pregnancies. While the pathogenesis of preeclampsia remains unclear, it is known that placenta-derived factors trigger the disease by activating maternal endothelial cells prior to the onset of clinical symptoms. Extracellular vesicles (EVs) of different sizes extruded by the placenta may be one factor. The truncated/secreted form of Flt-1 (sFlt-1) has also been implicated in the pathogenesis of preeclampsia. We investigated whether placental EV production is altered in preeclampsia such that they induce endothelial cell activation, and whether (s)Flt-1 is involved.

**Methods:**

Macro-, micro-, and nano-vesicles were collected from normal and preeclamptic (PE) placental explants, and separated by differential centrifugation. The number and size of micro- and nano-vesicles was measured by nanoparticle tracking analysis and their ability to activate endothelial cells was quantified by endothelial cell intercellular adhesion molecule 1 expression and monocyte adhesion. The levels of Flt-1 were measured by western blots and ELISA.

**Results:**

PE placentae extruded significantly more micro- and nano-vesicles than control placentae and the extruded micro-vesicles were larger than those from control placentae. Micro- and nano-vesicles from both first trimester and term human placentae carried Flt-1 and levels were significantly increased in EVs from severe, but not mild, PE compared to normotensive placentae. All fractions of EVs from PE placentae activated endothelial cells, and for micro- and nano-vesicles, activation was reduced in the presence of exogenous vascular endothelial growth factor (VEGF), a Flt-1 neutralizing antibody, or by pre-treatment with VEGF. While EV-bound VEGF constituted over 20% of the total detected VEGF secreted by PE and normotensive placentae, EV-bound Flt-1 did not significantly contribute to the total level of sFlt-1/Flt-1 released by human third trimester placentae.

**Discussion:**

Micro- and nano-vesicles extruded by human placentae carry Flt-1 across gestation and in severe preeclampsia, the levels of vesicle-bound Flt-1 are upregulated. All fractions of PE placental EVs activated endothelial cells and for micro- and nano-vesicles, this was in part due to the ability of EV-bound Flt-1 to sequester VEGF. That placental EVs can activate endothelial cells supports the contention that EVs are one placental toxin contributing to the pathogenesis of preeclampsia.

## Introduction

Preeclampsia is a hypertensive disease unique to human pregnancy that causes significant maternal and fetal mortality and morbidity worldwide (1, 2). Preeclampsia is diagnosed by *de novo* hypertension after 20 weeks of gestation accompanied by a range of other clinical signs/symptoms, such as proteinuria. Endothelial cell dysfunction is a central hallmark of preeclampsia that occurs prior to the onset of clinical signs (3–6). Preeclampsia can be classified into early onset (<34 weeks at diagnosis) or late onset disease, as well as severe (>160 mmHg systolic blood pressure or >110 mmHg diastolic blood pressure) and mild forms (7). Different subtypes of preeclampsia may have different etiologies, pathogenesis, and clinical outcomes (2).

While the pathogenesis of preeclampsia remains poorly understood, the placenta is known to play a crucial role as this disease can only occur in the presence of a placenta or a placental tumor (8, 9). Furthermore, delivery of the placenta is the only definitive treatment currently available for preeclamptic (PE) pregnancies. One vasoactive factor that has been proposed to play a central role in the pathogenesis of preeclampsia is soluble fms-kinase 1 (sFlt-1) (10, 11). Fms-kinase 1 is a cell-surface receptor for vascular endothelial growth factor (VEGF) and placental growth factor (P*l*GF), two important vascular growth factors that mediate angiogenesis and vascular homeostasis. The truncated version of Flt-1 is a soluble molecule that consists only of the ligand binding domain, allowing sFlt-1 to bind to VEGF/P*l*GF without downstream effects, effectively sequestering the growth factors (12).

A landmark study by Maynard et al. demonstrated that the injection of sFlt-1 into pregnant rats induced hypertension, proteinuria, and glomerular endotheliosis, features observed in women with preeclampsia (10). Furthermore, in clinical studies, the level of circulating sFlt-1 has also been reported to be increased in women with preeclampsia, and a reduced ratio of circulating P*l*GF:sFlt-1 has been proposed to be a potential predictor of subsequent preeclampsia (13–15).

In addition to the secretion of soluble factors, the placenta also releases a large range of extracellular vesicles (EVs) into the maternal circulation (16). These vesicles include syncytial nuclear aggregates and mononuclear trophoblasts (collectively termed macro-vesicles in this study), as well as subcellular micro- and nano-vesicles, including exosomes (17, 18). The release of macro-vesicles has been reported to be increased in preeclampsia over 60 years ago (19–21), and more recently, the number of micro-vesicles produced by PE placentae have also been reported to be increased, especially in early onset disease (22–26).

Our lab has previously shown that in contrast to macro-vesicles from normotensive placentae, macro-vesicles extruded from PE placentae can activate endothelial cells (27). Interestingly, Flt-1 is a component of the protein cargo of all fractions of EVs (macro-, micro-, and nano-vesicles) extruded by normal first trimester human placentae (28). Furthermore, increased Flt-1 has been detected in STBMs (a mixture of micro- and nano-vesicles) derived from perfused PE placentae, compared to normotensive placentae (29).

Therefore, in this study, using a minimally disruptive placental explant model, we collected macro-, micro-, and nano-vesicles from PE and normotensive placentae and characterized the size and number of micro- and nano-vesicles extruded. Whether different size fractions of EVs carry Flt-1 in preeclampsia and can activate endothelial cells through a Flt-1-mediated mechanism was also investigated. Finally, how much vesicle-bound Flt-1 contributes to the total level of (s)Flt-1 released from the placenta, and the levels of Flt-1 in different-sized vesicles from the same first trimester human placentae was determined.

## Materials and Methods

### Sample Collection

The use of human placentae collected following surgical termination of pregnancy from Epsom Day Unit, Greenlane Hospital (New Zealand) or following delivery from Auckland City Hospital (New Zealand) was approved by the Auckland Regional Health and Disabilities Ethics Committee. All subjects gave written informed consent in accordance with the Declaration of Helsinki.

The diagnosis of preeclampsia was based on the observation of new onset hypertension (>130/100 mmHg on two occasions separated by 4 h) and proteinuria (>300 mg protein within 24 h) after 20 weeks of gestation (7). Severe preeclampsia was defined as systolic blood pressure over 160 mmHg or diastolic blood pressure over 110 mmHg (7). Table 1 summarizes the clinical information of patients recruited for this study.

**Table 1:** Clinical characteristics of recruited patients.

	Controls (*n* = 10)	Mild PE (*n* = 5)	Severe PE (*n* = 9)
Maternal age	33.3 ± 2.0	35.0 ± 1.4	34.8 ± 1.6
Gestation at diagnosis (weeks)	–	36.3 ± 1.1	31.2 ± 1.4
Gestation at sampling (weeks)	39.0 ± 0.6	36.5 ± 1.1	33.9 ± 1.1*
Systolic BP (mmHg)	119.1 ± 4.9	150.2 ± 2.4*	169.2 ± 5.1*
Diastolic BP (mmHg)	74.6 ± 2.5	89.2 ± 1.3*	103.2 ± 3.2*
Proteinuria	–	>1+*	>1+*
Fetal weight (g)	3431 ± 150	3415 ± 180	1599 ± 233*

*p < 0.05 compared to controls (unpaired *t*-tests, mean ± SEM).

### Collection of Placental EVs

Placental EVs were collected from human placentae using a well-established *ex vivo* placental explant culture method as previously described (28, 30). Briefly, 400 mg explants were dissected from the villous placenta and cultured in Netwell^TM^ inserts in Advanced DMEM/F12 medium supplemented with 2% FBS and 1% Penicillin/Streptomycin (Invitrogen). After 18 h of culture at 37°C in 5% CO_2_/95% air, the culture medium was aspirated and placental EVs of different sizes were separated by sequential centrifugation at 4°C at 2,000 *g* for 5 min (macro-vesicle fraction), 20,000 *g* for 1 h (micro-vesicle fraction), and 100,000 *g* for 1 h (nano-vesicle fraction) (Avanti J30I Ultracentrifuge, JA 30.50 Ti fixed angle rotor, Beckman Coulter). Contaminating red blood cells were removed from the macro-vesicle fraction by hypotonic lysis in ultrapure water (EMD Millipore) and contaminating leukocytes were depleted using anti-CD45 magnetic beads (Dako).

### Nanoparticle Tracking Analysis

Micro- and nano-vesicles from 1 g of placental tissue were resuspended in 0.2 μm-filtered PBS (Sigma-Aldrich) to reach measurable concentrations in the range of 2 to 15 × 10^8^ vesicles/mL before analysis on an NS300 Nanosight instrument (Nanosight). All automatic settings were applied with viscosity setting at 0.95 cP and temperature at 25°C. A single measurement entailed three 30-s videos, and each sample was measured thrice at camera level 10. The detection threshold was also set at 10, and subsequent analysis of data was performed using the NTA3.0 software (Nanosight).

### Protein Extraction and Western Blotting

Total proteins from placental EVs were extracted using RIPA buffer (50 mM Tris, 150 mM NaCl, 1% sodium deoxycholate, 0.1% SDS, 1% Nonidet P40 substitute, 1 mM PMSF, pH7.4) supplemented with protease inhibitor (Roche) and resolved by SDS-PAGE. Proteins were transferred onto Hybond™-C extra nitrocellulose membranes (Amersham Biosciences) which were blocked for non-specific binding by incubating in 5% non-fat milk powder/PBST for an hour at 20°C. Membranes were then probed with rabbit anti-human Flt-1 (Abcam, 1:1,000) or mouse anti-human β-actin (Abcam, 1:5,000) antibodies before applying HRP-conjugated anti-mouse/rabbit IgG antibodies (Jackson ImmunoResearch, 1:2,000). Target proteins were visualized using Amersham™ ECL™ Prime detection reagent on an Image Quant LAS3000 (GE Healthcare). Images were annotated using Adobe^®^ Photoshop^®^ Elements 5.0.

### Cell Culture

The human microvascular endothelial cell line (HMEC-1) was purchased from ATCC (CRL3243) and cultured in MCDB-131 medium supplemented with 10% FBS, 1% l-Glutamine, and 1% Penicillin/Streptomycin (Invitrogen). Human U937 monocytes were purchased from ATCC (CRL1593.2) and cultured in Advanced DMEM/F12 medium supplemented with 2% FBS and 1% Penicillin/Streptomycin (Invitrogen). Cells were cultured at 37°C in 5% CO_2_/95% air.

### Endothelial Cell Activation

#### Cell-Surface ELISA to Quantify ICAM-1 Levels

The increase in the expression of intercellular adhesion molecule 1 (ICAM-1) on the endothelial cell surface is a well-established marker of endothelial cell activation (31, 32). Six thousand HMEC-1 cells were grown to confluence in each well of a 96-well plate before exposure to placental EVs in quadruplicate, with and without exogenous VEGF supplementation (100 ng/mL), a Flt-1 neutralizing antibody (10 µg/mL) or both treatments, for 24 h in quadruplicate. Then, cells were washed and cell-based ELISA using a mouse anti-human ICAM-1 antibody (MCA1135, Bio-rad, 1:100) was performed as previously described (32). o-Phenylenediamine was used as the substrate for detection at 490 nm using an xMark spectrophotometer (Bio-Rad).

#### Monocyte Adhesion Assay

After confluent HMEC-1 cells were exposed to placental EVs in quadruplicate for 24 h at 37°C, cells were washed and CellTracker™ Red CMTPX-labeled U937 monocytes were added (5 × 10^3^ monocytes/well of a 96-well plate). Monocytes and HMEC-1 cells were co-cultured for 5 h at 37°C. Then, unbound monocytes were thoroughly washed off with PBS and the CMTPX fluorescence remaining in each well was measured using a Synergy 2 microplate reader (BioTek) at 530/620 nm.

### ELISA

Commercial sandwich ELISA kits were used to quantify the levels of sFlt-1 and VEGF present in the placental explant culture medium. To collect placental conditioned medium, one 400 mg explant of PE or control placentae was cultured for 18 h as described above and after mixing, 1 mL of the conditioned medium was collected and divided into two portions: one of which was stored at −80°C without further processing while the other portion was centrifuged at 100,000 *g* for 1 h to deplete EVs. The resulting supernatant was stored at −80°C until analysis.

All ELISA assays included a standard curve of known concentrations of the target protein and were performed according to the manufacturer’s instructions in duplicate. The ELISA kit used to measure sFlt-1 levels (ALX-850-264, Sapphire Bioscience) has a limit of detection of 30 pg/mL and an intra-assay and inter-assay coefficients of variation of 5.5 and 5.1%, respectively. The ELISA kit used to measure VEGF levels (ab100662, Abcam) has a limit of detection of 10 pg/mL and an intra-assay and inter-assay coefficients of variation of <10 and <12%, respectively.

### Statistical Analysis

Statistical differences were examined using parametric or non-parametric *t*-tests or one-way ANOVA with Dunn’s multiple comparisons test as appropriate on GraphPad PRISM 6.01 (GraphPad Software Inc.). The statistical test used for each dataset is noted in the figure legends. An adjusted *p* value <0.05 was considered statistically significant.

## Results

### PE Placentae Released Significantly More Micro- and Nano-vesicles Than Normotensive Placentae, and the Extruded Micro-vesicles Were Larger in Size

**Figure 1:**
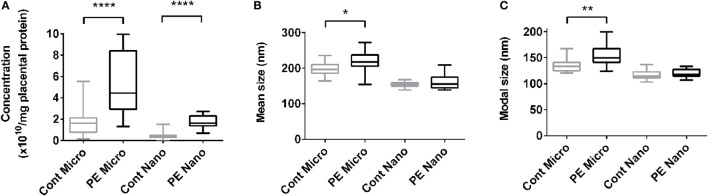
Production of micro- and nano-vesicles by PE placentae. Box and whisker graph demonstrating the amount of micro- and nano-vesicles extruded from normotensive (cont, *n* = 10) or preeclamptic (PE, *n* = 8) placental explants, normalized to the protein content of the originating placental explant **(A)**, and their mean **(B)** and modal **(C)** size. As data was not normally distributed, data is plotted as the median, interquartile range and range; and a Mann–Whitney test was performed to examine statistical differences between control and PE groups (*p < 0.05, **p < 0.01, ****p < 0.0001).

The increased production of macro-vesicles by PE placentae is well documented (19, 20, 33). In order to investigate whether the placental production of either micro- or nano-vesicles is affected in preeclampsia, micro- and nano-vesicles were collected from PE and normotensive placentae and their average sizes and concentrations were quantified by nanoparticle tracking analysis. Compared to normotensive control placentae, PE placentae produced significantly more micro- and nano-vesicles (*p* < 0.0001, *n* = 8, Figure 1A), and the mean and modal size of the extruded micro-vesicles, but not nano-vesicles, were also significantly increased (*p* < 0.05, *n* = 8, Figures 1B,C).

### All Fractions of EVs Extruded by PE Placentae Activated Endothelial Cells

**Figure 2:**
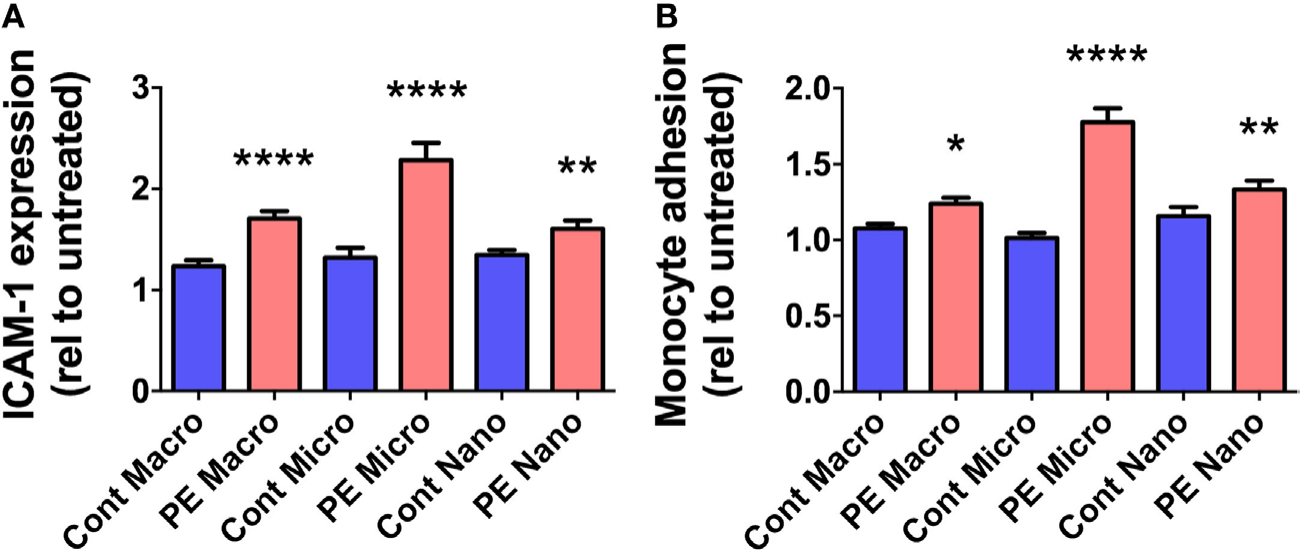
Levels of surface intercellular adhesion molecule 1 (ICAM-1) expression and monocyte adhesion by human microvascular endothelial cell line (HMEC-1) cells after co-culture with preeclamptic (PE) EVs. Human microvascular endothelial cells were co-cultured with macro-, micro-, and nano-vesicles from normotensive (cont, *n* = 6) and PE (*n* = 6) placentae for 24 h. Endothelial cell activation was quantified by measuring surface ICAM-1 expression **(A)** and monocyte adhesion **(B)**. Values were normalized to that of untreated HMEC-1 cells, and data are shown as mean ± SEM (*p < 0.05, **p < 0.01, *****p < 0.0001, unpaired *t*-test).

In order to investigate whether EVs extruded from PE placentae can activate endothelial cells, cell-based ELISA of endothelial surface ICAM-1 expression and monocyte adhesion assays were performed after exposure to macro-, micro-, and nano-vesicles from PE or control placentae for 24 h. All three sizes of EVs extruded from PE placentae significantly increased endothelial surface ICAM-1 expression (*p* < 0.01, *n* = 6, Figure 2A) and monocyte adhesion (*p* < 0.05, *n* = 6, Figure 2B) compared to the corresponding EV fractions from normotensive control placentae.

### Flt-1 Is Present in Placental EVs throughout Gestation

**Figure 3:**
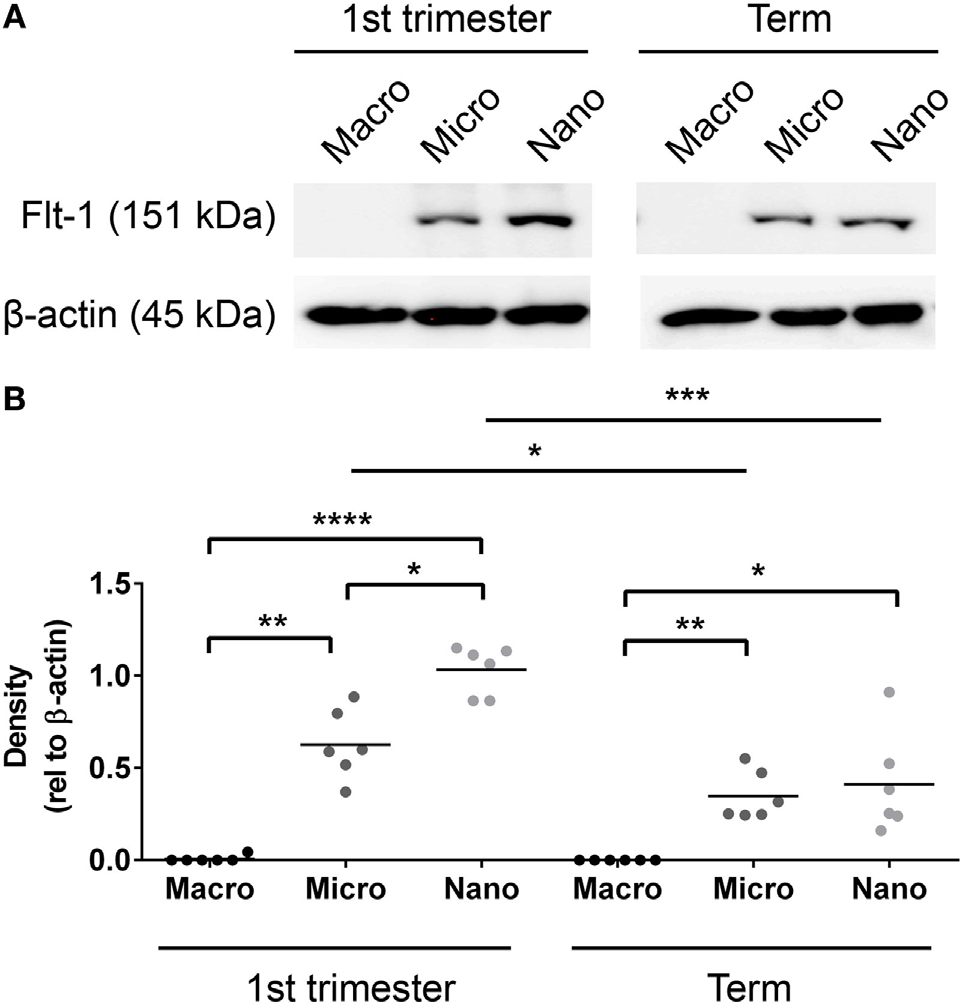
Levels of Flt-1 carried by EVs extruded from first trimester and term placentae. Total protein was extracted from macro-, micro-, and nano-vesicles collected from six first trimester and six term placentae. The presence of Flt-1 was detected by western blotting **(A)** and the levels of Flt-1 were semi-quantified relative to β-actin by densitometric analysis **(B)**. The mean value in each group is indicated (*p < 0.05, **p < 0.01, ****p < 0.0001, unpaired *t*-test).

Since Flt-1/sFlt-1 has previously been implicated in the pathogenesis of preeclampsia, whether Flt-1 is present in placental EVs at different gestations was investigated by western blotting. Flt-1 was readily detected in micro- and nano-vesicles from six first trimester placentae but only in macro-vesicles from one of six placentae (Figure 3). The levels of Flt-1 relative to β-actin were significantly higher in nano-vesicles compared to micro-vesicles (*p* < 0.01, *n* = 6, Figure 3) obtained from the same first trimester placenta. Flt-1 was also detected in micro- and nano-vesicles from normal term human placentae but was not detected in macro-vesicles (*n* = 6, Figure 3).

### Flt-1 Levels Are Increased in Micro- and Nano-vesicles from Severe PE Placentae

**Figure 4:**
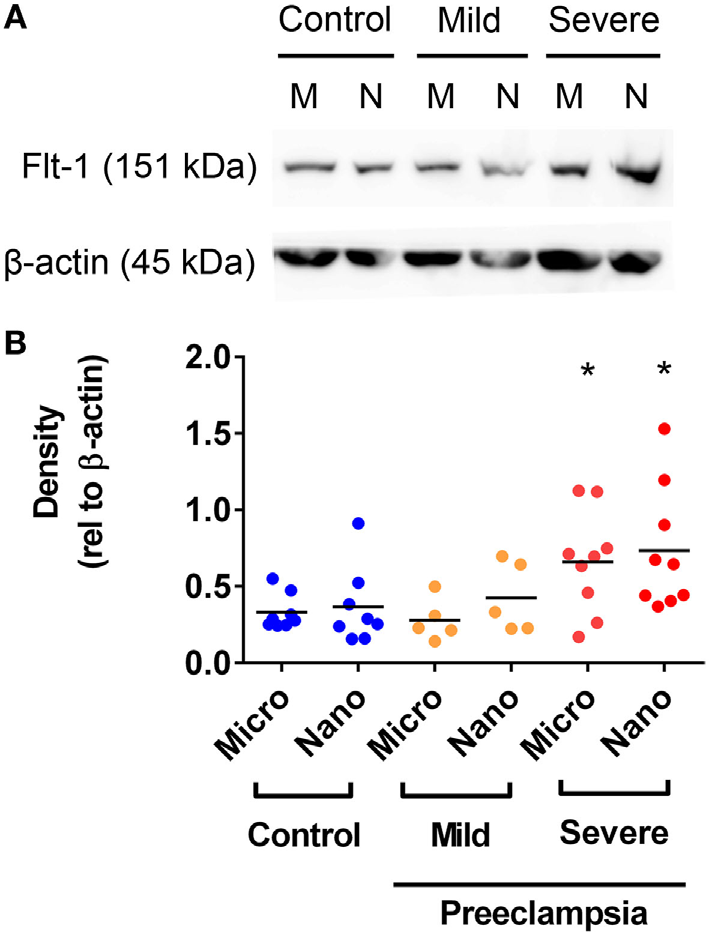
Levels of Flt-1 carried by micro- and nano-vesicles extruded from preeclamptic (PE) and normotensive placentae. The levels of Flt-1 present in micro- (M) and nano- (N) vesicles derived from normotensive (*n* = 10), mild PE (*n* = 5) and severe PE (*n* = 9) placentae were investigated by western blotting **(A)**. Densitometric analysis was performed relative to β-actin with the mean values as indicated [**(B)**, *p < 0.05, unpaired *t*-test].

In order to further investigate whether the levels of Flt-1 are altered in EVs extruded from PE placentae, Flt-1 levels in macro-, micro-, and nano-vesicles from five mild PE, nine severe PE, and ten normotensive placentae were semi-quantified by western blotting and densitometry. Flt-1 was not detected in macro-vesicles from PE or control near-term placentae (data not shown). However, the levels of Flt-1, relative to β-actin, were significantly higher in both micro- and nano-vesicles from severe PE placentae, compared with that from normotensive or mild PE placentae (*p* = 0.0184 for micro-vesicles, *p* = 0.0415 for nano-vesicles, *n* = 9, Figure 4). The levels of Flt-1 in micro- and nano-vesicles from placentae affected by mild preeclampsia were not significantly different to those from normotensive placentae (Figure 4).

### Flt-1 on Micro- and Nano-vesicles from PE Placentae Can Sequester VEGF, Contributing to Endothelial Cell Activation

Vesicle-associated Flt-1 may sequester free VEGF and have consequences for maternal endothelial cells. In order to investigate whether micro- and nano-vesicles extruded from PE placentae activate endothelial cells through a Flt-1 mediated mechanism, HMEC-1 cells were cultured with PE micro- or nano-vesicles, in the presence and absence of either 100 ng/mL exogenous VEGF, a Flt-1 neutralizing antibody (10 µg/mL), or both treatments before measuring surface ICAM-1 expression.

**Figure 5:**
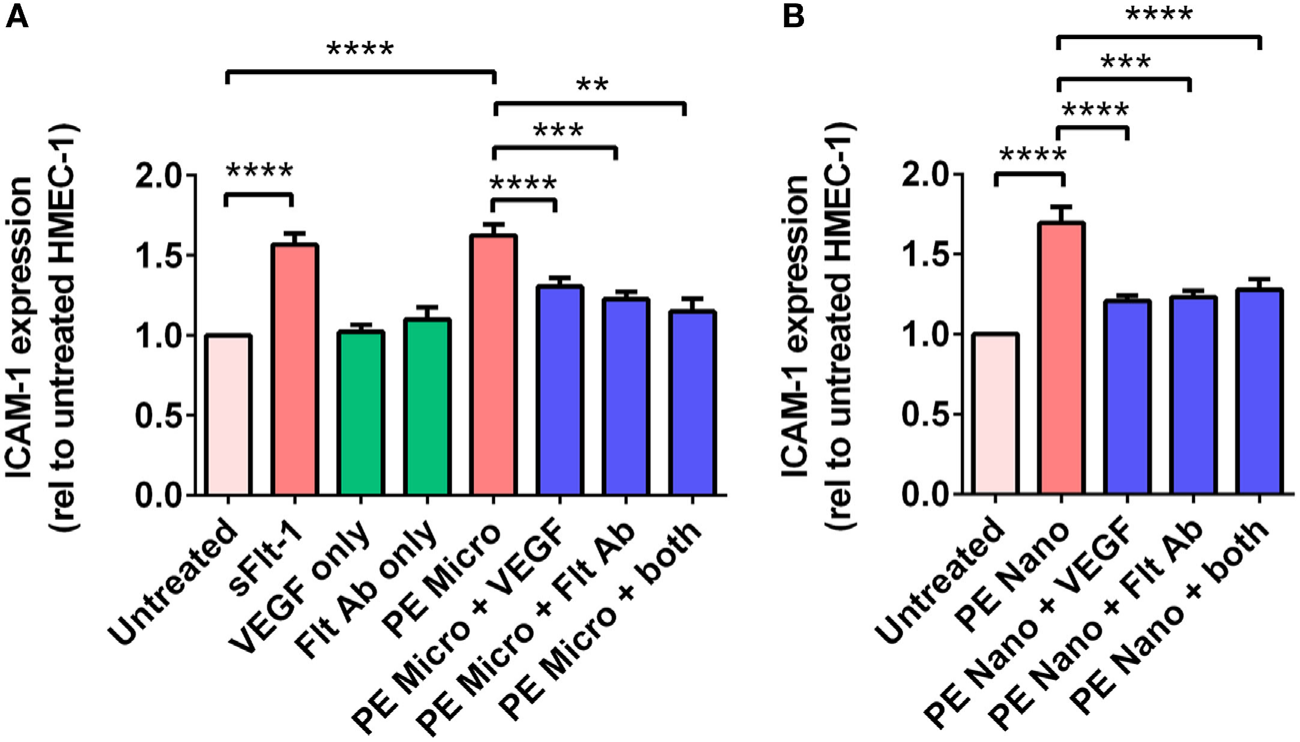
Levels of intercellular adhesion molecule 1 (ICAM-1) expression by human microvascular endothelial cell line (HMEC-1) cells after co-culture with preeclamptic (PE) micro- and nano-vesicles with and without vascular endothelial growth factor (VEGF) and Flt-1 neutralizing antibody. Micro- **(A)** and nano- **(B)** vesicles derived from PE placentae (*n* = 7) were added to HMEC-1 cells in the presence or absence of recombinant VEGF (100 ng/mL) or Flt-1 neutralizing antibody (10 µg/mL, Flt Ab) or both, in quadruplicate. Human microvascular endothelial cells were also cultured with sFlt-1 (200 pg/mL) as a positive control. After 24 hours, surface ICAM-1 expression was measured by cell-based ELISA. Data are presented relative to basal ICAM-1 expression by untreated HMEC-1 cells (mean ± SEM, **p < 0.01, ***p < 0.001, ****p < 0.0001, paired *t*-test).

Culture of HMEC-1 cells with 200 pg/mL of recombinant sFlt-1 significantly increased ICAM-1 expression (*p* < 0.0001, *n* = 5, Figure 5A), as did culture with PE micro- and nano-vesicles (*p* < 0.0001, *n* = 7, Figure 5). Compared to HMEC-1 cells cultured with PE micro-vesicles only, surface ICAM-1 expression was significantly reduced in the presence of recombinant VEGF (50% reduction, *p* < 0.0001) or the Flt-1 neutralizing antibody (35% reduction, *p* = 0.0002, *n* = 7, Figure 5A). Similarly, compared to HMEC-1 cells cultured with PE nano-vesicles only, endothelial cell-surface ICAM-1 expression was significantly reduced in the presence of exogenous VEGF (70% reduction, *p* < 0.0001) or the Flt-1 neutralizing antibody (67% reduction, *p* = 0.0005, *n* = 7, Figure 5B). For both placental micro- and nano-vesicles, the simultaneous addition of both recombinant VEGF and the Flt-1 neutralizing antibody did not have an additive protective effect (Figure 5).

#### Mechanisms of Interaction between VEGF and PE Placental EVs

**Figure 6:**
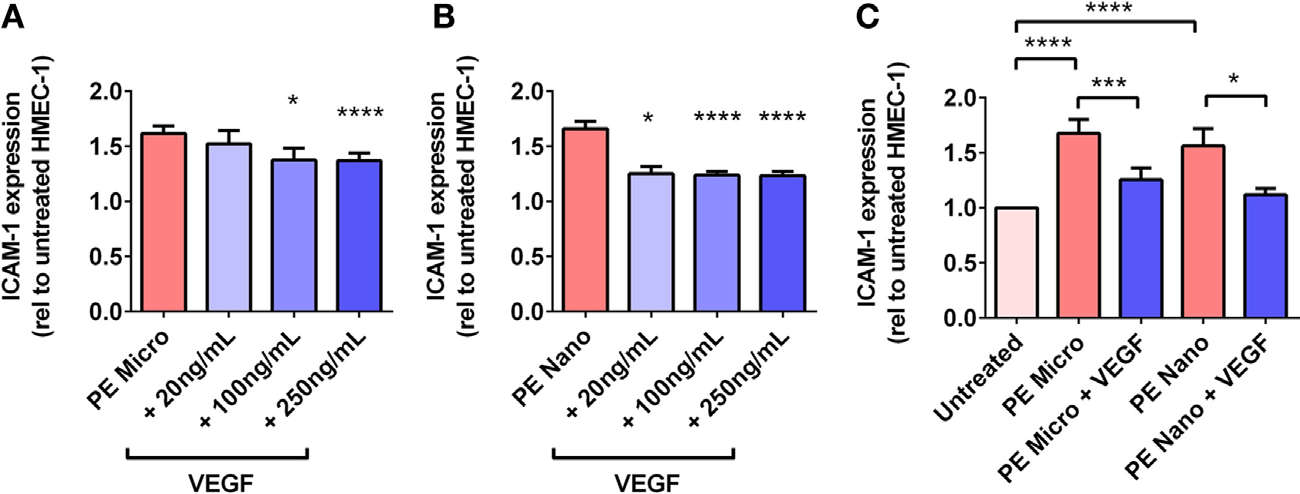
Levels of intercellular adhesion molecule 1 (ICAM-1) expression by human microvascular endothelial cell line (HMEC-1) cells after co-culture with preeclamptic (PE) micro- and nano-vesicles, in the presence of different VEGF treatment regimens. Human microvascular endothelial cells were cultured with PE micro- **(A)** or nano- **(B)** vesicles in the presence of 0, 20, 100, or 250 ng/mL exogenous VEGF, or PE vesicles that have been pretreated with 100 ng/mL VEGF prior to exposure to HMEC-1 **(C)**. After 24 h, surface expression of ICAM-1 was quantified and normalized to basal ICAM-1 levels expressed by untreated HMEC-1 cells (mean ± SEM, *p < 0.05, ***p < 0.001, ****p < 0.0001, paired *t*-test).

For PE micro-vesicles, the protective effect of exogenous VEGF was concentration-dependent from 100 ng/mL to 250 ng/mL (*n* = 3, Figure 6A), while for PE nano-vesicles, the protective effect of exogenous VEGF was also concentration dependent but started at a lower concentration of 20 ng/mL (*n* = 3, Figure 6B). In order to confirm that the protective effect of exogenous VEGF against endothelial cell activation induced by PE micro- or nano-vesicles was due to quenching of the Flt-1 present on these EVs, micro- and nano-vesicles were collected from PE placentae and divided into two portions. One portion was incubated in culture medium while the other portion was incubated with recombinant VEGF (100 ng/mL) for 15 min at 20°C. Both portions were then ultracentrifuged to remove any excess free VEGF before adding to HMEC-1 cells. After 24 h, endothelial cell-surface ICAM-1 expression was measured. Compared to HMEC-1 cells cultured with the untreated PE micro-vesicles, HMEC-1 cells cultured with PE micro-vesicles that have been pre-treated with recombinant VEGF had significantly lower levels of cell-surface ICAM-1 expression (75% reduction, *p* = 0.0007, *n* = 3, Figure 6C). Similarly, PE nano-vesicles that have been pre-treated with recombinant VEGF induced significantly lower levels of HMEC-1 cell-surface ICAM-1 expression compared to the untreated PE nano-vesicles (72% reduction, *p* = 0.0307, *n* = 3, Figure 6C).

### PE Placentae Secreted More sFlt-1 Than Control Placentae, but Levels Were Not Significantly Affected by Depletion of EVs

**Figure 7:**
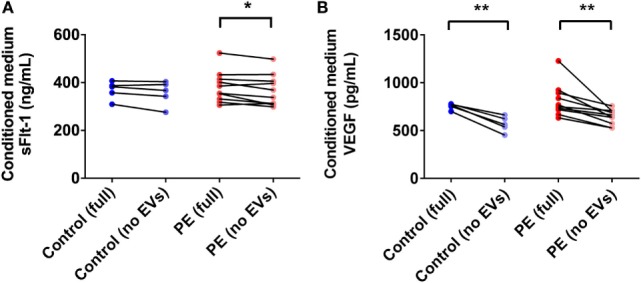
Levels of sFlt-1 and vascular endothelial growth factor (VEGF) measured in the conditioned medium from preeclamptic (PE) and normotensive placentae, before and after ultracentrifugation. Conditioned media were collected from normotensive (control) and PE placentae (full). Then, half of the conditioned medium was ultracentrifuged at 100,000 × *g* for 1 h to deplete EVs (no EVs) and the levels of sFlt-1 **(A)** and VEGF **(B)** in the no EVs and full conditioned media samples were quantified by sandwich ELISA (*p < 0.05, **p < 0.01, unpaired *t*-test between control and PE data, paired *t*-test within control or PE groups).

In order to determine the total amount of sFlt-1 produced by PE placentae and how much of this was bound to EVs, placental conditioned medium was collected from PE and normotensive placentae, and divided into two portions. One half was immediately centrifuged at 100,000 × *g* for 1 h to deplete EVs while the other half was not processed further. Once all samples had been collected, an ELISA for sFlt-1 was performed. The mean concentration of sFlt-1 in the conditioned medium from PE placentae before ultracentrifugation was 12.5 ± 0.3 ng/mL (mean ± SEM), which was significantly higher than that from normotensive placentae (10.2 ± 0.4 ng/mL; *p* = 0.0013, *n* = 6, Figure 7A). Depletion of EVs by ultracentrifugation reduced 6.8% of the total sFlt-1 measured from PE placentae (from 12.5 ± 0.3 ng/mL to 11.7 ± 0.3 ng/mL, *n* = 6, Figure 7A), and 0% of the total sFlt-1 measured from normotensive control placentae (10.2 ± 0.4 ng/mL and 10.3 ± 0.2 ng/mL, *n* = 5, Figure 7A).

### Vesicle-Associated VEGF Accounts for over 20% of the Total VEGF Released by the Placenta

One of the major agonists for Flt-1 is VEGF. In order to determine whether the placental production of VEGF is altered in preeclampsia, and whether the produced VEGF is vesicle-associated, conditioned media from PE and normotensive control placentae were collected and processed as described above for sFlt-1. The mean level of VEGF measured in the conditioned media from PE and control placentae before ultracentrifugation were not significantly different (PE: 815 ± 54 pg/mL, control: 768 ± 5.4 pg/mL, mean ± SEM, *p* > 0.05, *n* = 10, Figure 7B). Depletion of EVs by ultracentrifugation significantly reduced the measured levels of VEGF in both PE and control conditioned media samples by 21 and 26%, respectively (PE: 642 ± 25 pg/mL, control: 568 ± 36 pg/mL, *p* < 0.01, *n* = 10, Figure 7B).

## Discussion

Preeclampsia is a life-threatening disease of pregnancy with unknown causes and a complex pathogenesis. Much recent research has focused on investigating soluble factors that may contribute to the development of this disease, however, EVs released by the placenta may also play a role. It has long been known that excess macro-vesicles are produced by PE placentae (19, 20) and recently, increases in the production of micro- and nano-vesicles have also been reported in preeclampsia (23, 26). Our observations corroborate these findings, showing that not only do PE placentae produce significantly more micro- and nano-vesicles than normotensive placentae, the extruded micro-vesicles were significantly larger in size. This suggests that in preeclampsia, there is a change in the process leading to extrusion of micro-, but not nano-, vesicles; reinforcing the concept that despite being produced by the same cell (the syncytiotrophoblast), these vesicles have different biogenic origins and may therefore have differing effects on maternal physiology.

In this study, we showed for the first time that all size fractions of EVs extruded by PE placentae can activate endothelial cells, and that for micro- and nano-vesicles, this was in part due to the increased level of Flt-1 present. Furthermore, while PE placentae secreted more sFlt-1 than control placentae, vesicle-associated Flt-1 did not contribute significantly to the total level of secreted Flt-1. In contrast, the major ligand of Flt-1, VEGF, was also produced by the human placenta and over 20% was vesicle-associated. Finally, it was interesting to observe that micro- and nano-vesicles from first trimester human placentae also carried Flt-1, whereas free serum sFlt-1 levels are reportedly very low in the first trimester (11, 34).

Multiple studies have previously reported that PE placentae extrude more macro-vesicles/syncytial nuclear aggregates (19, 21, 33) and micro-vesicles (22, 23, 26) than normotensive placentae. Our work corroborates those studies and adds that PE placentae also extruded increased levels of nano-vesicles compared to normotensive placentae. This increase in EV production may be reflective of altered cell death or function of the syncytiotrophoblast during preeclampsia (35–38). This suggestion is supported by reports showing intrinsic differences in the expression of transcription factors that regulate trophoblast differentiation between normal and PE placentae (39).

Using an *ex vivo* explant culture model and sequential centrifugation, we were able to compare the micro- and nano-vesicles extruded by each placenta and show that in addition to releasing increased numbers of both micro- and nano-vesicles, PE placentae also extruded larger micro-vesicles, while the average size of nano-vesicles remained unchanged. This novel observation is intriguing as it has been suggested that larger vesicles are more pro-inflammatory compared to smaller vesicles (40). Therefore, in addition to an increased circulating load of placental EVs in preeclampsia, micro-vesicles produced by PE placentae are also potentially more proinflammatory, which may contribute to the pathogenesis of this disease. That we did not also observe an increase in the average size of nano-vesicles extruded by PE placentae supports the notion that micro- and nano-vesicles are produced through different intracellular mechanisms. For example, it is well-established that exosomes, one type of nano-vesicles, are produced specifically through a regulated endosomal pathway (41, 42). In contrast, micro-vesicles are thought to be produced via budding of the plasma membrane.

In addition to the quantitative changes in EV production in preeclampsia, all size fractions of EVs from PE placentae significantly increased endothelial cell activation, while EVs from normotensive placentae did not. This supports and expands on our previous findings that macro-vesicles from PE placentae can induce endothelial cell dysfunction (27). Interestingly, several older studies have reported that micro-vesicles isolated from normotensive term placentae can also affect endothelial cell function and reduce their viability (29, 43, 44). In contrast, in this work, we did not observe significant activation of endothelial cells by EVs from normal placentae. These discrepancies are likely a result of the different experimental procedures used to obtain placental micro-vesicles, since mechanical disruption has been reported to yield EVs with higher lipid content than vesicles isolated from placental explant culture or perfusion methods, contributing to their ability to cause endothelial cell dysfunction (45). Therefore, using a minimally disrupted placental explant culture model, we contribute to the growing body of evidence that placental EVs can interact with endothelial cells, and that while EVs from PE placentae deleteriously affected endothelial cell function, EVs from normotensive placentae appeared well-tolerated. These findings support the hypothesis that EVs may be one placental toxin/danger signal that can contribute to the pathogenesis of preeclampsia.

It has been previously reported that increased Flt-1 is present in STBM (mixtures of placental micro- and nano-vesicles) isolated from perfused PE placentae (29). Our findings extend upon that finding to show that the levels of Flt-1 carried by both micro- and nano-vesicles derived from cultured severe PE placentae were significantly higher than that from normotensive placentae. In contrast, we did not detect Flt-1 in macro-vesicles derived from normal term placentae, although Flt-1 has previously been reported on syncytial nuclear aggregates derived from PE placentae that were washed *ex vivo* (46).

Vesicle-associated Flt-1 may exist in several conformations in relation to the vesicle, including being transmembrane or within the vesicle. If vesicle-associated Flt-1 is orientated with the ligand-binding domain facing the external environment, then it is likely that vesicle-associated Flt-1 will have the same function as sFlt-1 and be able to bind and sequester free VEGF and P*l*GF in the maternal circulation. Thus, micro- and nano-vesicles from PE placentae could act as “sponges” for VEGF and P*l*GF in the circulation, preventing downstream interactions between these angiogenic factors and target cells, such as endothelial cells. This idea was first hypothesized by Tannetta and colleagues, who showed that STBM collected by mechanical dissection of normal healthy term placentae carried Flt-1 with the ligand-binding domain orientated outside of the vesicle and can bind VEGF (29). However, as previously cautioned, micro-vesicles collected by mechanical dissection of the placenta do not mimic the physiological process of vesicle release *in vivo* and these EVs can cause overt cell death due to changes in their cargo (45, 47). Furthermore, different disease states may also affect the packaging of cargo into EVs (48, 49). Thus, it is crucial to characterize the orientation and function of Flt-1 directly in PE micro- and nano-vesicles in order to definitively determine whether vesicle-associated Flt-1 play any role in the pathogenesis of preeclampsia. Our placental explant culture model produces EVs that do not induce endothelial cell death (50) and is more likely to be physiologically relevant than the mechanical disruption model (45, 47). Thus, using EVs collected from cultured PE placentae in combination with microvascular endothelial cells, we showed that pre-treatment of PE micro- and nano-vesicles with VEGF prior to co-incubation with endothelial cells significantly reduced the ability of PE micro- and nano-vesicles to activate endothelial cells. These findings support that at least some, if not all, vesicle-associated Flt-1 is positioned with the ligand-binding domain facing the external environment, allowing vesicle-associated Flt-1 to sequester VEGF and induce endothelial cell dysfunction.

However, it should be noted that while PE macro-vesicles do not carry Flt-1, they can still activate endothelial cells. This clearly demonstrates that placental EVs carry more than one “danger signal”/toxin that can affect maternal endothelial cells. For example, we have recently reported that PE macro-vesicles carry increased levels of IL-1β and HMGB1 that can act in distinct pathways to Flt-1 to cause endothelial cell activation (27, 51). Similarly, none of the vesicle fractions from mild preeclamptic placentae contained elevated levels of Flt-1, yet all fractions of PE EVs (regardless of the severity of disease) caused endothelial cell activation. Due to the limited sample sizes in this study, we were unable to subdivide our functional assays into mild and severe PE groups to determine whether the extent of the effects were different between disease subtypes. This is an important question to answer in the future as different subtypes of preeclampsia have significant differences in their etiology, pathogenesis and clinical outcomes.

We have previously reported that placental EVs carry a large range of different proteins (28). While the majority of proteins were shared between different-sized vesicles, each fraction also carried proteins that were unique. Those observations, combined with our present results regarding Flt-1, demonstrate that it is important to study the full range of EVs extruded from the PE placenta when considering the overall contribution of placental EVs to preeclampsia. Furthermore, in contrast to classic endocrine factors, EVs contain a large abundance of other factors that are important in the targeting of the vesicle and the rate of interaction with recipient cells (28, 52). Thus, the presence and level of Flt-1 alone does not necessarily correlate with the ability of a vesicle to activate endothelial cells. Rather, the milieu of factors present in/on each vesicle is likely to act together to produce a net effect on recipient cells. This again reinforces the importance to study all of the placental EVs extruded in order to understand their contribution to the pathogenesis of preeclampsia.

Since increased levels of Flt-1 were detected on micro- and nano-vesicles extruded from PE placentae, we were interested to investigate what contribution vesicle-associated Flt-1 made to the total level of apparent sFlt-1 produced by the placenta. Previous studies have reported that PE placentae produced significantly higher levels of sFlt-1 compared to normotensive placentae (46, 53). This observation is supported by our work showing that PE placentae produced 22% more sFlt-1 than normotensive placentae. Despite the increased levels of Flt-1 on micro- and nano-vesicles from PE placentae, vesicle-associated Flt-1 accounted for less than 7% of the total sFlt-1 secreted by PE placentae. Nevertheless, EV-associated Flt-1 may still be an important contributor to the pathogenesis of preeclampsia because: (1) we have shown that increased numbers of micro- and nano-vesicles are extruded by PE placentae and (2) we have previously reported that different size fractions of EVs are targeted to specific maternal organs such as the kidneys and liver, organs that are affected in preeclampsia (52, 54). Therefore, while increased levels of free sFlt-1 may mediate global endothelial cell dysfunction by sequestering circulating VEGF, vesicle-associated Flt-1 may be concentrated in specific organs such that even a low circulating level of vesicle-associated Flt-1 may have a large biological significance by causing organ-specific endothelial cell damage, such as glomerular endotheliosis which is commonly reported in women with preeclampsia (3, 6). It is interesting to speculate that the different distribution patterns of placental EVs, and therefore vesicle-bound Flt-1, *in vivo* may contribute to the wide variety of organ-specific symptoms observed between different women with preeclampsia.

In this work, we also investigated the secretion of VEGF by cultured PE placentae. Despite several studies reporting that the levels of VEGF are increased in the placental tissue in preeclampsia (55, 56), our findings suggest that the secretion of VEGF by the placenta is not altered in preeclampsia. In combination with the increased placental production of sFlt-1 observed in preeclampsia, this supports that there is a shift toward more anti-angiogenic factors being released by the placenta during preeclampsia. Interestingly, in contrast to Flt-1/sFlt-1 which was not predominantly vesicle-associated, more than 1/5th of the VEGF secreted by the human placenta was vesicle-associated and able to be removed by ultracentrifugation. While beyond the scope of this work, whether this vesicle-associated VEGF is complexed to the Flt-1 on the EVs warrants further investigation since the presence of Flt-1/VEGF complexes on micro- and nano-vesicles may affect the manner in which these vesicles interact with the maternal vasculature. That a significant portion of circulating VEGF is vesicle-associated may explain why previous investigations of VEGF as a potential biomarker of preeclampsia have not been consistent, as differences in sample preparation may have affected the levels of EVs present. For example, it has been reported that micro-vesicles are present in plasma but not serum (57).

In this work, Flt-1 was also detected in micro- and nano-vesicles extruded from first trimester human placentae, with significantly higher levels in nano-vesicles than micro-vesicles. Conversely, the levels of Flt-1 in micro- and nano-vesicles derived from normal term human placentae were similar. This is the first time that the levels of Flt-1 in different fractions of placental EVs have been compared and these observations suggest that different intracellular mechanisms regulate the packaging of Flt-1 into different vesicle fractions. Furthermore, this regulation may also change with gestation. That vesicle-associated Flt-1 levels are different at different gestational ages introduces a caveat to our work since it is essentially impossible to obtain gestation-matched normal control placentae for early onset preeclampsia. Thus, gestational age mismatch may potentially explain some of the differences in the levels of vesicle-associated Flt-1 observed in severe preeclampsia. That Flt-1 is present on EVs extruded from first trimester placentae supports the idea that vesicle-associated Flt-1 may potentially contribute to the early pathogenesis of preeclampsia from the first trimester onward.

In summary, we have shown that not only are more micro- and nano-vesicles extruded by PE placentae compared to normotensive control placentae, the micro-vesicles extruded are larger in size, which may indicate that they are more proinflammatory. Furthermore, all size fractions of EVs from PE placentae activated endothelial cells *in vitro*, and for micro- and nano-vesicles, this was in part mediated by increased levels of Flt-1 which can sequester VEGF. While vesicle-associated Flt-1 accounts for less than 10% of the total sFlt-1 secreted by PE placentae, since EVs are targeted to a limited number of maternal organs, vesicle-associated Flt-1 may be concentrated, and play a greater role, in specific maternal organs. Conversely, a significant portion of the VEGF secreted by PE placentae was vesicle-associated, suggesting that the *in vivo* mechanisms for the placental release of these two vasoactive factors into the maternal circulation are different. Since placental micro- and nano-vesicles carry Flt-1 from the first trimester onward, placental EVs may be one placental toxin/danger signal that contributes to the pathogenesis of preeclampsia, partially in a Flt-1-dependent manner.

## Ethics Statement

Use of human placentae collected following surgical termination of pregnancy from Epsom Day Unit, Greenlane Hospital (New Zealand) or following delivery from Auckland City Hospital (New Zealand) was approved by the Auckland Regional Health and Disabilities Ethics Committee. All subjects gave written informed consent in accordance with the Declaration of Helsinki.

## Author Contributions

PS provided the placental samples and clinical background of the study. MT and QC conducted the experiments. MT, QC, JJ, and LC interpreted the results. MT drafted the manuscript. All authors contributed to study design and approval of the final manuscript.

## Conflict of Interest Statement

The authors declare that the research was conducted in the absence of any commercial or financial relationships that could be construed as a potential conflict of interest.
